# Quantifying maternal antibody transfer to colostrum and cord blood reveals virus-specific selectivity in dogs

**DOI:** 10.3389/fimmu.2025.1753521

**Published:** 2026-01-14

**Authors:** Serena S. Teh, Lotta H. Truyen, Sarah Woodyear, Jessica Palmeri, Ana Alice Pimenta-Pereira, Alexis A. E. Peprah, Ella Lanman, Tom M. Lonergan, Abigail Reid, Soon Hon Cheong, Sarah L. Caddy

**Affiliations:** 1 Baker Institute for Animal Health, Cornell University, Ithaca, NY, United States; 2 University of Veterinary Medicine Hannover, Hannover, Germany; 3 Department of Microbiology and Immunology, College of Veterinary Medicine, Cornell University, Ithaca, NY, United States; 4 Department of Clinical Sciences, College of Veterinary Medicine, Cornell University, Ithaca, NY, United States

**Keywords:** anti-viral antibodies, canine, colostrum, IgG, maternal antibody, umbilical cord, canine parvovirus, canine distemper virus

## Abstract

**Background:**

Neonatal infections are a leading cause of mortality in dogs, with up to 30% of puppies dying within the first 3 weeks of life. During this critical period of immune development, protection is highly dependent on maternal antibodies (MatAbs) transferred across the placenta and via colostrum. Despite the critical importance of this transfer, limited information is available regarding the biological or clinical factors that determine its magnitude, whether specific antibodies are preferentially transferred, or how these processes vary across a broad population of dogs.

**Methods:**

To quantify and explore the determinants of MatAb transfer in dogs, we analyzed matched maternal serum, cord blood, and colostrum samples collected from 44 client-owned dams undergoing cesarean section at a university veterinary hospital. Total immunoglobulin G (IgG) and virus-specific antibodies against canine parvovirus (CPV) and canine distemper virus (CDV) were analyzed. We also evaluated the influence of maternal factors, including age, breed, body weight, parity, and litter size, on MatAb transfer efficiency.

**Results:**

Across this diverse population, we observed limited transplacental transfer of MatAbs (4.5%–6% of the maternal titer), consistent with previous studies and as expected given the endotheliochorial placenta of dogs. In contrast, virus-specific IgG was highly enriched in colostrum, with 10.7-fold (CPV) and 8.1-fold (CDV) increases relative to serum. Transfer efficiency was significantly greater for virus-specific antibodies than for total IgG (3.2-fold), suggesting selective enrichment of antiviral antibodies during colostrogenesis. Maternal serum antibody titer emerged as the primary factor influencing antibody transfer efficiency.

**Conclusions:**

These findings provide the most comprehensive quantification to date of MatAb transfer routes in dogs, revealing preferential transfer of virus-specific IgG to colostrum and highlighting the crucial role of colostrum intake in neonatal immunity. This work establishes a foundation for identifying antibody characteristics that influence MatAb transfer efficiency and reinforces the importance of ensuring that dams have adequate titers of virus-specific IgG prior to breeding.

## Introduction

Neonatal infections are a leading cause of puppy mortality, with young animals especially vulnerable while their immune systems are still developing. Mortality within the first 3 weeks of life can reach up to 30%, and a proportion of these deaths is attributable to early infections (1, 2). To compensate for this period of immunodeficiency, maternal antibodies (MatAbs), sometimes referred to as maternally derived antibodies, are transferred to pups from the dam across the placenta and via colostrum. Previous studies have shown that inadequate antibody transfer is associated with significantly increased puppy mortality (1).

The dominant route of MatAb transfer varies across mammalian species. In humans, the majority of maternal immunoglobulin G (IgG) received by infants is transferred across the placenta. In contrast, in ruminants and horses, all maternal IgG is transferred via colostrum. Dogs represent an intermediate species; it has previously been estimated that 5%–10% of total maternal IgG crosses the placenta (3, 4). This difference is attributed to variation in placental structure among species: three tissue layers separate the maternal and fetal circulations in the human hemochorial placenta, whereas four tissue layers are present in the canine endotheliochorial placenta and six layers in the epitheliochorial placenta of ruminants and horses (5). The majority of MatAb is transferred in dogs, therefore via colostrum (6, 7). However, previous studies quantifying MatAb transfer routes have notable limitations; most have examined only a small number of research dogs (4, 8, 9) and quantified either total IgG or IgG specific to a single pathogen.

In this study, we sought to quantify MatAb transfer across the placenta and via colostrum in a diverse group of client-owned dogs. To obtain samples from dogs at birth, we recruited cases undergoing cesarean section at a university hospital. This approach enabled the collection of cord blood from puppies, which we have demonstrated to be a valuable proxy for maternal IgG transferred across the placenta. We also collected the first colostrum from the dam and obtained matched dam serum samples. Samples from this unique cohort were comprehensively analyzed by enzyme-linked immunosorbent assay (ELISA)for MatAbs specific to two viruses to which neonates are highly susceptible: canine parvovirus (CPV) and canine distemper virus (CDV) (10, 11). These results were compared with total IgG titers and revealed an unexpected preferential transfer of antiviral antibodies relative to total IgG. Overall, this analysis enabled precise quantification of MatAbs across the placenta and mammary gland in dogs, facilitated exploration of factors influencing canine MatAb transfer, and reinforced the critical importance of colostrum ingestion for puppies. Collectively, these findings highlight the dog as a powerful comparative model for investigating MatAb transfer mechanisms and virus-specific antibody selectivity, enabling cross-species comparisons of conserved MatAb features among mammals.

## Methods

### Sample collection

To study MatAbs, samples were collected from 44 client-owned dogs undergoing cesarean sections at the Cornell University Hospital for Animals (CUHA, IACUC approval 2023-0003). Maternal blood was collected from either the cephalic or saphenous vein. Umbilical cord blood samples were taken immediately after the surgical delivery of pups, as follows: the umbilical cord was clamped at either end to detach the placenta from the puppy. Whenever possible, a syringe and needle were used to aspirate the blood from the cord. If this method was not feasible, the clamp closer to the puppy was removed, and a 1.5-ml microcentrifuge tube was used to collect cord blood manually expressed from the blood vessels. Samples of both live births and stillborns were collected and used in the analysis. Colostrum was collected from at least four teats within 1 h of delivery, pooled, and stored in a single 1.5 ml microcentrifuge tube (variation has been reported between teats (7)). All samples were kept at 4°C for up to 24 h until further processing, after which maternal and cord blood samples were centrifuged for 5 min at 8,000 rpm to separate serum. All samples were subsequently stored long term at − 80°C. Basic clinical and demographic data for each dam were recorded, including age, breed, weight, body condition score, parity, litter size, and any known vaccination history.

### Production of canine parvovirus virus-like particles

For the production of CPV virus-like particles (VLPs) required for ELISAs, the Bac-to-Bac Baculovirus Expression system was used in DH10 *Escherichia coli* bacteria. The plasmid encoding the CPV-VP2 capsid protein was purified using a commercial kit (Monarch Plasmid Miniprep Kit) according to the manufacturer’s recommendations. *Spodoptera frugiperda* (Sf9) insect cells were transfected with VP2 BacMid using Cellfectin-II transfection reagent (Invitrogen, California, USA), using 8 μl Cellfectin-II and 1 μg BacMid in 200 μl per T-25 flask with serum-free Grace Media. After incubating for 4 h at 27°C, the transfection mixture was removed, and Grace Media with 10% fetal calf serum (FCS) was added, followed by incubation for 4–7 days at 27°C. To harvest the P0 virus stock, the culture media were collected and centrifuged for 5 min, and then the cell culture was centrifuged for 5 min at 500 g. Subsequently, 200 μl of P0 viral stock was added to fresh cultures and incubated for 4–5 days in a humidified incubator at 27°C. To amplify the P1 virus in Sf9 cells, supernatant was collected from transfected Sf9 cells and centrifuged for 5 min at 500×*g*. A 100-μl aliquot of supernatant was then transferred to previously seeded Sf9 insect cells. Cells were incubated for 4–5 days in a humidified incubator at 27°C, after which the virus was harvested. Protein production was subsequently performed in Hi5 cells infected with P2 virus under the same conditions. VLPs were purified using a cesium chloride (CsCl) gradient, followed by dialysis to remove residual CsCl. VLP production was confirmed by SDS-PAGE with Coomassie staining and Western blotting. Protein concentrations were determined using both a bicinchoninic acid (BCA) assay and a Qubit fluorometric assay.

### Canine distemper virus propagation

Vero cells stably expressing canine signaling lymphocytic activation molecule [SLAM; also known as CD_150_, the receptor for CDV (12)] were incubated at 37°C and 5% CO_2_ in DMEM containing 50 IU/ml penicillin, 50 μg/ml streptomycin, and 5% FCS (complete media). Cells were infected with CDV (Onderstepoort strain, provided by Diego Diel, Cornell University) at a MOI of 0.01 in 5 ml of infection media with 2% FCS. The virus–media mix was incubated with cells for 1 h at 37°C with gentle side-to-side rocking every 15 min. Complete media was then added, and the flask was incubated and checked daily for cytopathic effect (CPE). Once CPE was observed, the supernatant was collected, centrifuged at 1,600 rpm for 5 min at 4°C, aliquoted, and stored at − 80°C. A TCID_50_ assay was used to determine viral titer. A total of 2 × 10^4^ Vero-dogSLAM cells were seeded per well of a 96-well plate in a total volume of 50 μl. A 10-fold dilution of the supernatant containing virus was prepared in complete media, and 50 μl per well of each dilution was added in quadruplicate to the seeded cells. The last row of wells remained uninfected as a negative control. The plate was incubated until a clear CPE was visible, and the titer was calculated using the Reed–Muench method.

### ELISAs

For virus-specific ELISAs, high-binding ELISA plates (Greiner, North Carolina, USA) were coated with either 15 ng of CPV-VLPs in phosphate-buffered saline (PBS) per well or 1 × 10^5^ TCID_50_ CDV particles. Plates were incubated overnight at 4°C, washed with PBS containing 0.1% Tween 20 (“PBS-T”), and blocked with 5% milk in PBS-T. After washing with PBS-T, serially diluted dam serum (1:800 starting dilution), cord serum (1:100 starting dilution), or colostrum (1:1,600 starting dilution) was added, followed by incubation for 2 h at 37°C. After washing with PBS-T, either anticanine IgG (H+L) conjugated to horseradish peroxidase (HRP) (Cat. No. PA1-29738, Invitrogen) or anticanine IgG2 (Cat. No. A40-121P, Bethyl Laboratories, Texas, USA) was added for 1 h. Following a final wash step, tetramethylbenzidine (TMB) substrate (Abcam, Massachusetts, USA) was added for 10 min. This reaction was stopped using 1 M H_2_SO_4_, and absorbance was measured at optical density, 450nm (OD450) (BioTek Cytation 7 Cell Imaging Multimode Reader, Agilent, California, USA). Each sample was tested in duplicate. Every plate included a positive control (pooled serum samples from five healthy, vaccinated dogs), and a virus-specific IgG-negative control (Marshall Bioresources, New York, USA) was included on every plate. To correct for background signal, the optical density (OD) values from wells coated with PBS only were subtracted from the OD values of wells coated with VLPs or virus. The corrected OD450 values were plotted against dilution in GraphPad Prism, and nonlinear regression analysis with interpolation was used to calculate endpoint titers. The endpoint titer was defined as the reciprocal of the dilution at which the OD450 crossed the detection threshold, calculated as the mean OD450 of all PBS-coated wells plus three standard deviations. Results were only accepted if the nonlinear regression fit for both the standard curve and the sample curve had a coefficient determination (*R*^2^) ≥ 0.9.

Indirect ELISA assays were developed in-house to quantify total IgG in clinical samples. Rabbit anticanine IgG (Cat. No. SA5-10309, Invitrogen) was coated onto ELISA plates at 1 μg/ml in PBS overnight at 4°C. Wells were washed three times with PBS-T and then blocked with 5% milk in PBS-T. After washing with PBS-T, serially diluted dam serum, cord serum, or colostrum was added. Each sample was tested in duplicate. For the standard curve, serially diluted canine IgG (Cat. No. 0129-01, Southern Biotech), starting at 5 μg/ml, was added in duplicate to the plate. Samples were incubated for 2 h at 37°C. After washing, the TMB substrate was added, and the reaction was stopped using 1 M H_2_SO_4_. Absorbance was measured at OD450. Background signal was corrected by subtracting PBS-only values, as performed for virus-specific ELISAs. Corrected OD450 values were plotted against the dilution factor using GraphPad Prism, and nonlinear regression analysis with interpolation to the standard curve was performed to calculate the IgG concentration in each sample. Results were accepted only if the nonlinear regression fit for both the standard curve and the sample curve had an *R*^2^ ≥ 0.9.

### Statistical analysis

Based on preliminary data and paired sample comparisons, recruitment of 25 dogs was estimated to provide 80% power (α = 0.05) to detect a standardized difference of 0.6 in antibody transfer ratios between maternal and neonatal samples, a magnitude comparable to that reported in human studies (13, 14). To ensure robust analysis across a naturally occurring cohort, matched samples were ultimately collected from 44 dams, exceeding the minimum requirement. Puppies from the same litter were treated as biological replicates, and data were aggregated at the litter level to avoid pseudo-replication and eliminate the need for hierarchical model analysis. All statistical analyses were conducted using GraphPad Prism (version 10.3.1). End-point titer outcomes of CPV- and CDV-specific IgG, as well as total IgG concentrations, were nonnormally distributed; therefore, all were analyzed using the Kruskal–Wallis test followed by Dunn’s multiple comparisons test. Transfer ratios between dam and colostrum IgG, and between dam and cord IgG, were analyzed using Mann–Whitney *U* tests. Correlation analyses included nonlinear regression, Pearson’s correlation for normally distributed variables, and Spearman’s rank correlation for nonnormally distributed variables. Statistical significance was defined as *p* ≤ 0.05 for all comparisons.

## Results

### Study population

A total of 44 female dogs (“dams”) presenting to Cornell University Hospital for Animals for cesarean sections were enrolled in this study. The dams had a mean age of 3.6 years old (range 1–8 years), and multiple breeds were represented, including brachycephalic breeds (American Bulldog [four], French Bulldog [eight], English Bulldog [two], bully cross [2], pitbull [1], Chihuahua [1], shih tzu cross [1]) and nonbrachycephalic breeds (labrador [four], corgi [three], Golden Retriever [three], Leonberger [two], others [12]). All cesarean sections were emergency procedures except for a single elective procedure. Demographic and clinical details for each dam are presented in **Table 1**.

**Table 1:** Maternal demographic and clinical details of the study cohort.

Range	Mean	Standard deviation	Factor	Cases
Age of dam (years)				
1–8	3.6	1.8	< 2 years	4 (9%)
			2–3 years	18 (41%)
			4–5 years	15 (34%)
			6 years+	7 (16%)
Litter size				
1–11	6.5	2.6	< 3	1 (2%)
			3–6	24 (55%)
			> 6	19 (43%)
Parity				
1–4	1.6	0.9	Primiparous	19 (43%)
			Multiparous	23 (52%)
			Unknown	2 (5%)
Breed				
			Brachycephalic	19 (43%)
			Non-brachycephalic	25 (57%)
Body condition score (1–5)				
1–5	3.3	0.6	≤ 2	2 (5%)
			2.5–3.5	30 (68%)
			≥ 4	9 (20%)
			Not recorded	3 (7%)
Vaccination status				
			Up to date	20 (45%)
			Not up to date	6 (14%)
			Unknown	18 (41%)

### Transfer of canine virus-specific maternal antibody

IgG is the major antibody isotype transferred from mother to offspring in all mammalian species (15, 16). For puppies, the transfer of MatAbs specific for life-threatening pathogens is critically important for neonatal health. Therefore, we first aimed to quantify IgG for two significant canine pathogens: CPV and CDV. We also reasoned that most dams would be seropositive to these viruses due to core vaccination recommendations (17); thus, studying the transfer of these MatAbs would be feasible. Virus-specific IgG was quantified in dam serum and compared with MatAbs detectable in colostrum. We further compared virus-specific IgG in dam serum with that detected in serum separated from cord blood, representing the first reported use of cord blood sampling to quantify MatAbs delivered transplacentally in dogs. This cord sampling strategy enabled precise measurement of placental antibody transfer, overcoming limitations of prior methods that relied on blood sampling from newborn puppies. Direct sampling of puppies is more ethically sensitive, technically challenging, and potentially confounded by early colostrum intake.

Virus-specific IgG titers for all samples in our cohort were quantified using in-house ELISAs. Serial dilutions of each sample were generated, and the endpoint titer was defined as the highest dilution at which antibody binding to the viral antigen remained detectable. Endpoint titers are presented as the reciprocal of this final positive dilution. A small number of dams had undetectable titers of IgG specific for CPV (three dams, 6.8%) or CDV (six dams, 13.6%), with two dams having no detectable IgG to both viruses (4.5% dams). Although the presence of seronegative individuals in our breeding cohort was a concern, these rates were lower than those reported in a previous US study of hospitalized dogs, which found 19% and 50% seronegativity for CPV and CDV, respectively (18). We omitted colostrum or cord samples from cases with undetectable virus-specific IgG from further study. Additionally, in a small proportion of cases, it was not possible to collect the full clinical dataset (dam serum, colostrum, cord blood). The number of samples studied for each virus-specific IgG is listed in **Table 2**.

**Table 2:** Virus-specific IgG quantified by ELISA.

Virus		IgG endpoint titer			Transfer ratio	
		Dam	Colostrum	Cord	To colostrum	To cord
Canine parvovirus (CPV)	Mean	47474	437154	1837	10.7	0.045
	Standard deviation	54254	522299	3031	8.0	0.057
	Number of samples analyzed	41	37	129	37	118
Canine distemper virus (CDV)	Mean	8968	54210	209	8.1	0.060
	Standard deviation	21533	88096	411	6.3	0.047
	Number of samples analyzed	38	36	119	35	50

Endpoint titers are presented as the reciprocal dilution at which antibody binding to antigen was detectable. Transfer ratios were calculated as the IgG endpoint titer in colostrum or cord divided by the corresponding IgG titer in the matched dam serum.

**Figure 1:**
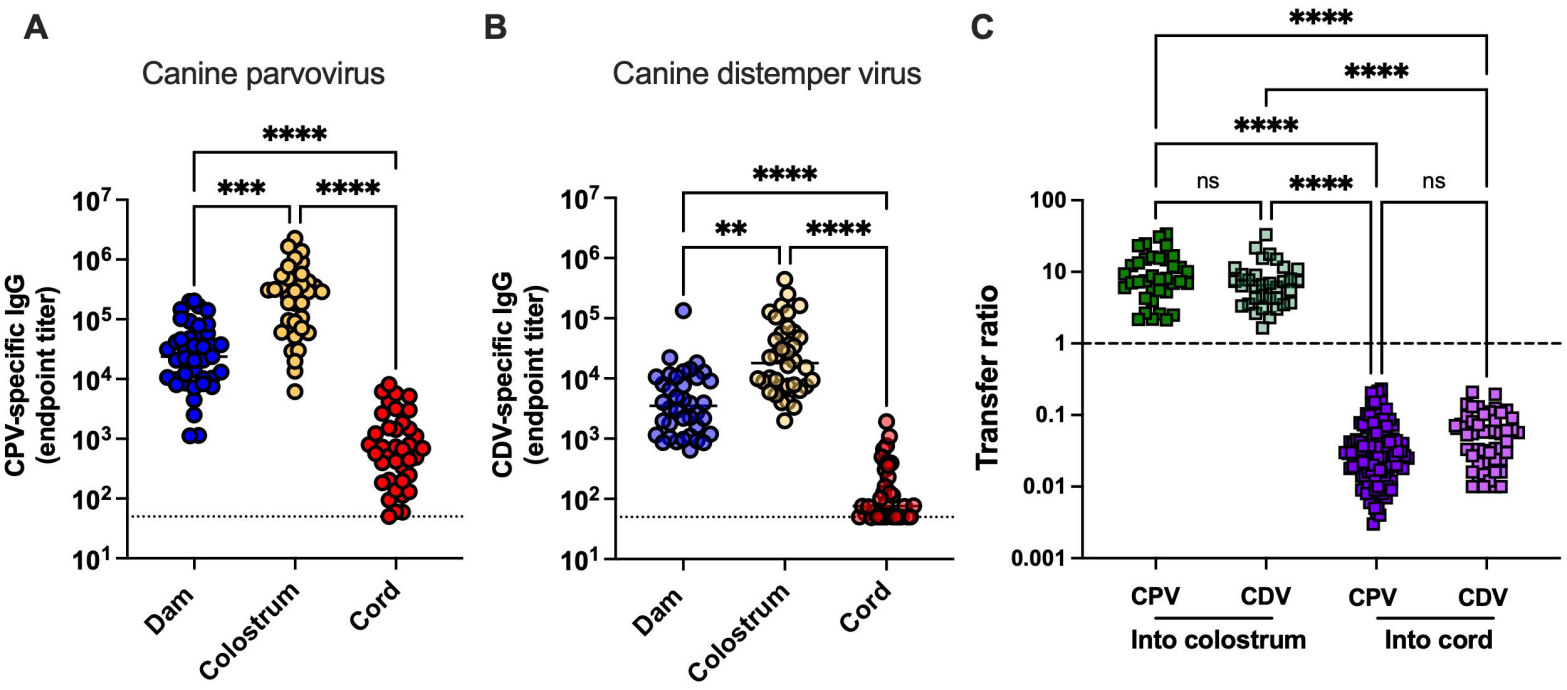
Virus-specific IgG quantified in dam serum, colostrum, and cord samples. Endpoint titers of IgG specific for **(A)** canine parvovirus (CPV) and **(B)** canine distemper virus (CDV) were quantified in clinical samples collected from dams and umbilical cords during cesarean sections. IgG transfer ratios between dam serum and colostrum/cord samples for puppies are shown in **(C)**. Dotted lines in **(A, B)** indicate the lower limit of quantification. The dashed line in **(C)** represents 100% transfer efficiency (equivalent IgG levels between samples). Statistical significance was assessed using Kruskal–Wallis tests with multiple comparisons. Asterisks denote statistical significance: ^**^p < 0.01, ^***^p < 0.001, and ^****^p < 0.0001.

IgG titers for each dam with detectable virus-specific IgG are presented in **Figures 1A, B**, with each point representing a single dam and her litter. The average (mean) cord IgG titer within each litter is shown as a single point. We found that virus-specific IgG in colostrum was significantly higher (*p* = 0.0003 and *p* = 0.0029, respectively, determined by Kruskal–Wallis test) than IgG circulating in dams for both viruses, as summarized in **Table 2**. In contrast, the mean cord IgG titer for each litter was significantly lower than that in the corresponding dam serum. To assess individual-level variation in MatAb transfer varied at the individual level, we calculated transfer ratios for every colostrum and cord sample for which the titer was above the lower limit of quantification. The results are presented in **Figure 1C**. This provides clear evidence that IgG is actively concentrated in the colostrum, resulting in levels that are 8.1-fold higher for CDV and 10.7-fold higher for CPV compared with those in the dam’s serum. Analysis of cord samples showed that approximately 5% virus-specific maternal IgG is able to cross the placenta.

Endpoint titers are presented as the reciprocal dilution at which antibody binding to the antigen was detectable. Transfer ratios were calculated as the IgG endpoint titer in colostrum or cord divided by the corresponding IgG titer in the matched dam serum.

To extend this analysis, we performed CPV-based ELISAs to quantify different MatAb isotypes and subclasses. Analysis of a subset of six groups of samples (dam, colostrum, cord) confirmed that IgM and IgA titers were very low in all sample types compared with the IgG isotype; therefore, further quantification of these isotypes was not pursued (data not shown). In several species, specific IgG subclasses are preferentially transferred to neonates (19, 20). However, in dogs, there is only limited analysis of the roles of different IgG subclasses in dogs at any life stage, and no prior studies have evaluated transfer rates of maternal IgG subclasses. To begin evaluating transfer differences, we adapted our in-house indirect CPV-specific IgG ELISA to quantify CPV-specific IgG2 antibodies. IgG2 has been reclassified as a combination of IgG subclass B and IgG subclass C (21); however, commercially available secondary antibodies cannot distinguish between these subsets (22). We measured CPV-specific IgG2 MatAbs in a randomly selected subset of samples (29 dams, with matched colostrum and cords) and demonstrated that there was no preferential transfer of CPV-specific IgG2 relative to all IgGs in our cohort (**Supplementary Figure S1**).

### Evaluation of total IgG in dam serum, colostrum, and cord samples

**Figure 2:**
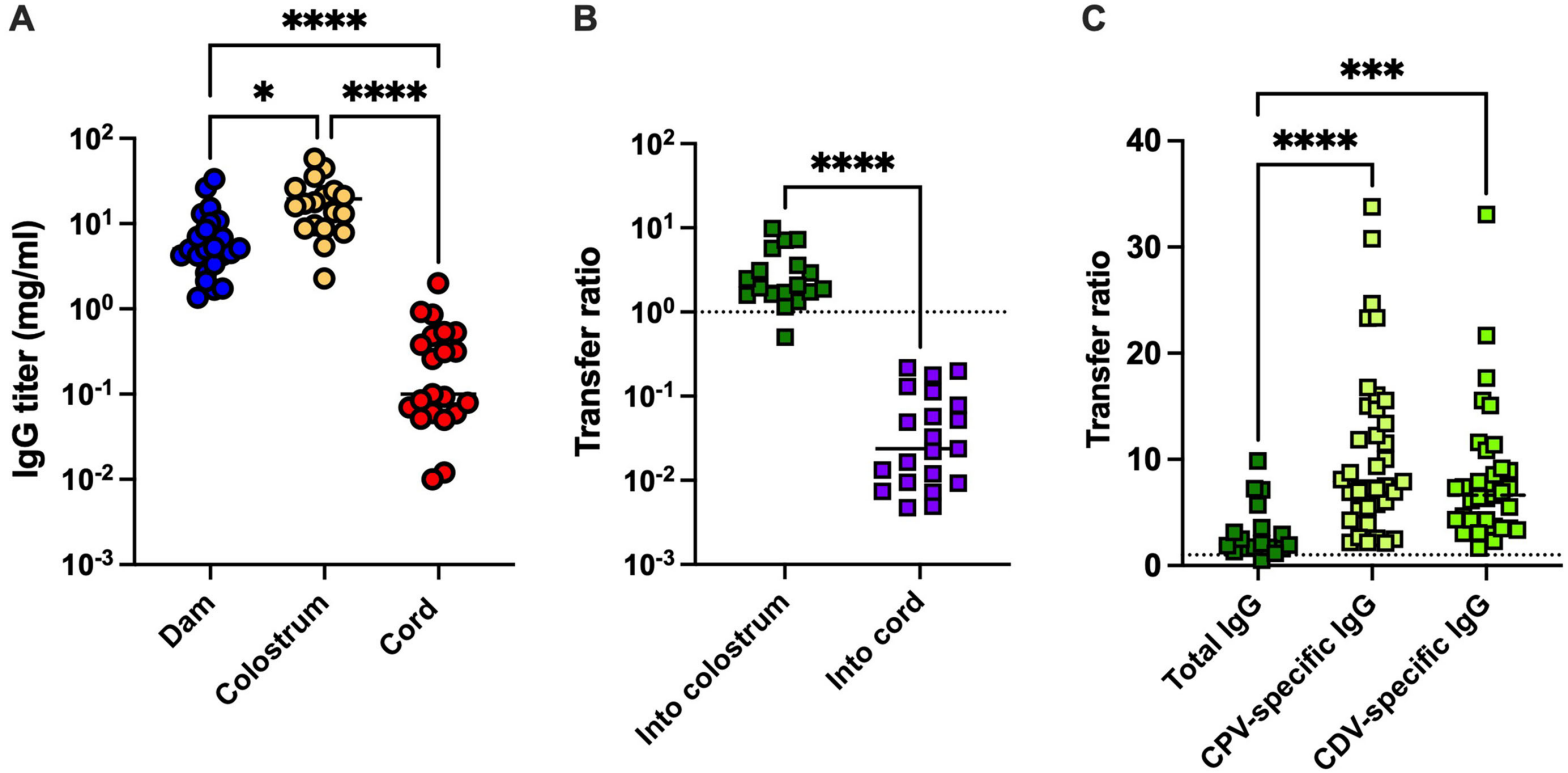
Total IgG titers quantified in dam serum, colostrum, and cord samples. **(A)** Total IgG titers were quantified in clinical samples by indirect ELISA; dam serum, *n* = 24; colostrum, *n* = 18; cord samples, *n* = 21 litters. **(B)** IgG transfer ratios via colostrum (*n* = 18) or cord (*n* = 21), calculated from total IgG titers. The dotted line indicates a transfer ratio of 1, representing equivalent IgG concentrations between samples. **(C)** Comparison of transfer ratios for total IgG and virus-specific IgG from maternal serum to colostrum. Statistical significance was assessed using a Kruskal–Wallis test with *post-hoc* multiple comparisons **(A, C)** and a Mann–Whitney *U* test **(B)**. Asterisks denote statistical significance: ^*^p < 0.05, ^***^p < 0.001, and ^****^p < 0.0001.

Previous studies of MatAb transfer have largely focused on total IgG rather than virus-specific MatAbs (7, 9, 23, 24). Therefore, we included total IgG quantification in our study to enable comparisons with both previous reports and our virus-specific MatAb analysis. IgG titers from 24 groups in the cohort (selected for having adequate remaining sample volume) are presented in **Figure 2A**, with each point representing the IgG titer of a single dam, a colostrum sample, or the average cord IgG titer within one litter. The mean total IgG titers in dam serum, colostrum, and cord were 7.9, 19.5, and 0.59 mg/ml, respectively. These findings closely match previously reported values, which quantified average total IgG in dam circulation as 8.1 mg/ml and in colostrum as 19.3–20.8 mg/ml (7, 24). Cord titers were also comparable to serum levels obtained from puppies at birth, reported as 0.3 mg/ml (25, 26) and 1.2 mg/ml (23). This confirms that umbilical cord blood is a viable, noninvasive alternative to direct neonatal blood sampling for evaluating the efficiency of placental IgG transfer.

Next, we calculated the transfer ratio of total IgG via colostrum and placenta, as shown in **Figure 2B**. Similar to virus-specific IgG, our results indicate that puppies receive the majority of maternally derived IgG through colostrum ingestion. Unexpectedly, the concentration of total IgG in colostrum relative to dam sera was significantly lower than that of virus-specific IgG, as shown in **Figure 2C**. These data suggest that virus-specific IgG is selectively transported into the colostrum for transfer to the puppies. In contrast, the transfer ratios from dam serum to cord for total IgG and virus-specific IgG were highly comparable (total IgG: 0.059, CPV-specific IgG: 0.045, and CDV-specific IgG: 0.060).

### Correlation between maternal antibody titers in dam, colostrum, and cord samples

Given the substantial variation in antibody titers in colostrum and cord samples relative to dam serum, we sought to identify which factors influence canine MatAb transfer. We first evaluated the relationship between IgG titers in dam serum and those in colostrum and cord samples. While studies in humans, sheep, and pigs have reported a positive correlation between maternal IgG titers and colostrum (27–29), previous studies in dogs did not identify such an association (7). Furthermore, multiple human studies have demonstrated a strong correlation between maternal and cord IgG titers, particularly for virus-specific antibodies (30–32), but this relationship has not previously been examined in dogs.

**Figure 3:**
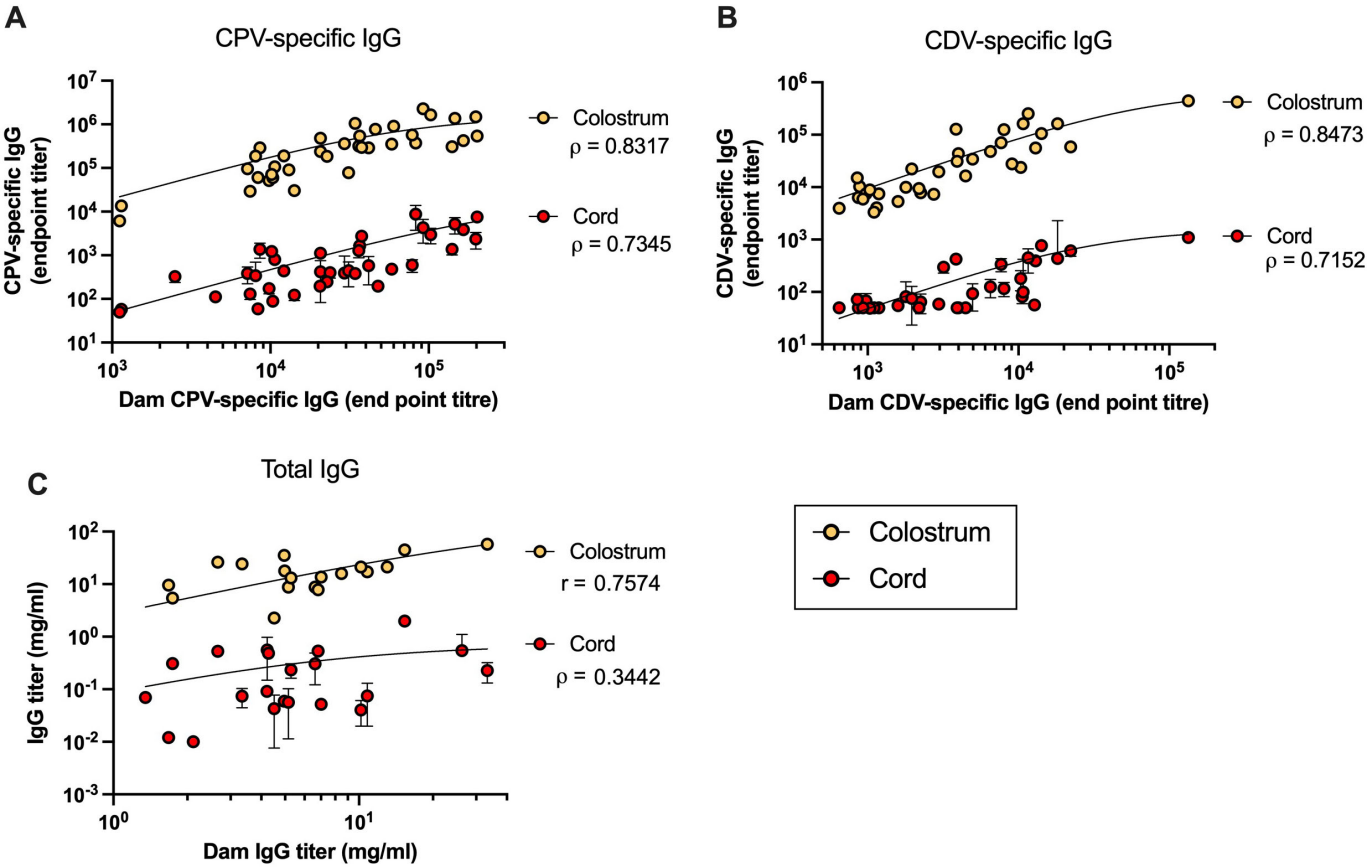
Correlation between dam IgG titer and IgG titers in colostrum and cord samples. Scatterplots of IgG titers for **(A)** CPV (colostrum, *n* = 36; cord, *n* = 38 litters) and **(B)** CDV (colostrum, *n* = 35; cord, *n* = 35 litters), and **(C)** total IgG (colostrum, *n* = 18; cord, *n* = 22 litters) as determined by ELISA. Nonlinear regression curves were fitted to the data. Statistical significance was assessed using Pearson’s correlation coefficient (*r*) for normally distributed data (total IgG colostrum) and Spearman’s rank correlation coefficient (*ρ*) for nonparametric data.

The correlations between dam serum IgG titers and cord or colostrum IgG titers for our two representative viruses are presented as scatterplots in **Figure 3**. Nonlinear regression analysis revealed a consistent positive trend across all virus-specific MatAb curves. Since the data were nonparametric, correlations were assessed using Spearman’s rank correlation coefficient (*r*), which demonstrated highly significant associations for each sample type and virus (*p* < 0.0001).

The strong association observed between dam and colostrum titers differed from the findings by Mila et al. (7). Notably, that earlier study quantified total IgG rather than virus-specific IgG in clinical samples. To assess whether antibody specificity influenced this relationship, we examined correlations between total IgG titers in each sample, as shown in **Figure 3C**. A moderate positive correlation was found between dam and colostrum total IgG titers (data normally distributed; Pearson’s correlation *r* = 0.7574, *p* = 0.0003), whereas no significant correlation was observed between dam and cord total IgG titers (data nonnormally distributed; Spearman’s correlation *r* = 0.3442, *p* = 0.1266).

### Correlation between MatAb transfer and maternal variables

**Figure 4:**
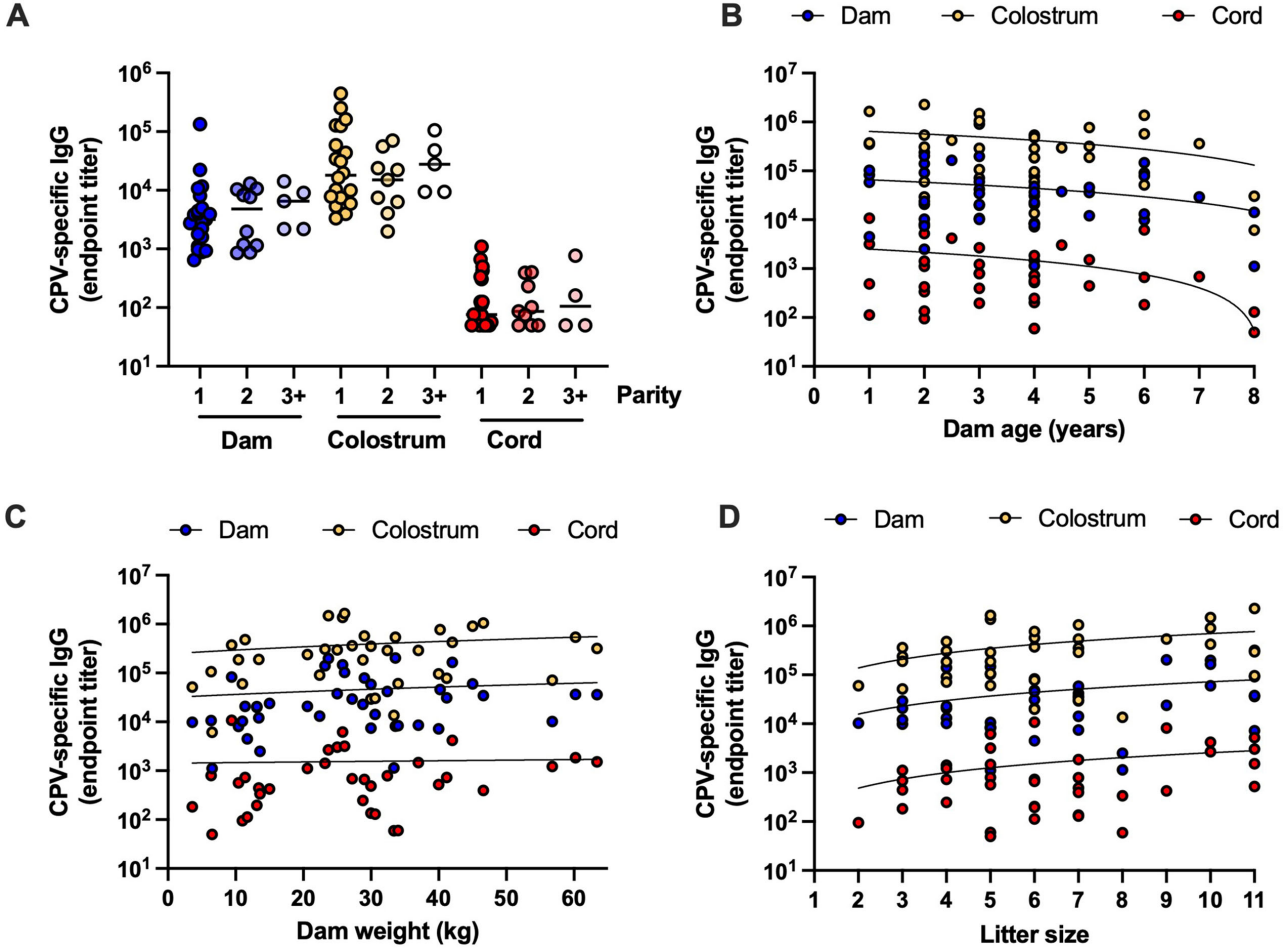
Correlation between CPV-specific IgG titers and biological variables. All values were determined using CPV-specific IgG ELISA. **(A)** Endpoint titers of CPV-specific IgG in dam serum, colostrum, and cord samples plotted against parity. The horizontal bar represents the mean titer for each group. **(B–D)** Endpoint titers of CPV-specific IgG plotted against dam age, dam weight, and litter size, respectively. Nonlinear regression curves were fitted to the data.

To identify any demographic or clinical factors associated with MatAb transfer, we analyzed the relationship between IgG titers in each sample and parity, dam age, dam weight, and litter size. **Figure 4** presents the results for CPV-specific IgG analysis, with comparable findings observed for CDV-specific IgG analysis (data not shown). Spearman’s rank correlation analysis revealed no significant associations between virus-specific IgG and any of these variables (*p* > 0.05). Vaccine status was also evaluated, but no differences were observed between dogs confirmed to be up to date with vaccinations (*n* = 20) and those not up to date (*n* = 6) (data not shown). A similar analysis using total IgG results (**Supplementary Figure S2**) likewise showed no correlations between MatAb levels and the factors examined.

### Heterogeneity in cord IgG titers and early protection from viral infection

**Figure 5:**
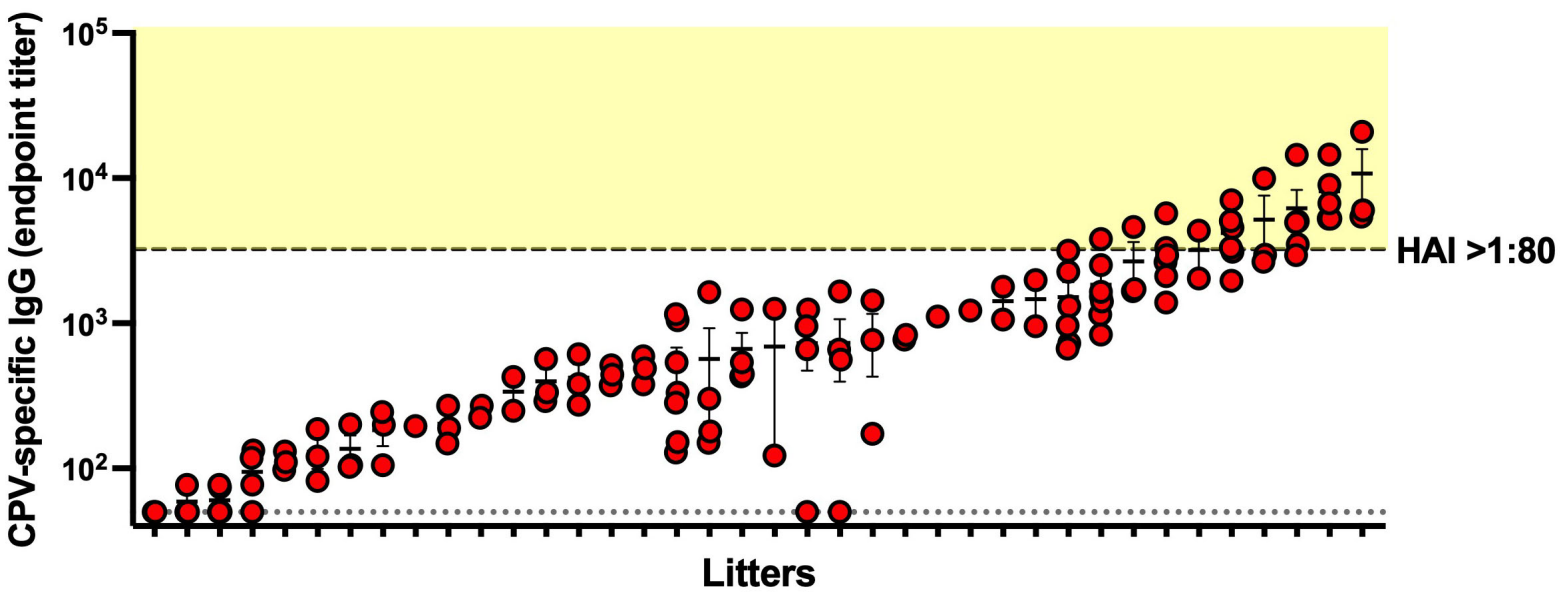
Titers of CPV-specific IgG in cord samples within each litter. Cord samples from each litter (*n* = 38 litters, one to seven cords per litter) are plotted together and arbitrarily ordered according to mean titer. Error bars represent the standard error of the mean. The dotted line indicates the lower limit of quantification of the ELISA assay. The dashed line corresponds to an ELISA endpoint titer of 3,239 and hemagglutination inhibition titer (HAI) > 1:80. The shaded yellow area represents protective IgG titers.

This study is the first to report analysis of MatAb titers in canine cord blood samples. As shown in **Figure 1**, approximately 5% of circulating virus-specific IgG in the dam is transferred across the placenta. Considerable variation was observed across litters, prompting an investigation into intralitter variation in IgG titers. **Figure 5** presents the CPV-specific cord IgG titers of 122 puppies from 38 litters. Of the six litters for which no cord results were available, three were from dams lacking CPV-specific IgG, and the remaining three had insufficient or uncollected samples. Although most litters had cord IgG titers that clustered closely together, nine litters exhibited a standard deviation > 1,000.

It was apparent that a proportion of puppies were born with moderately high levels of CPV-specific IgG. We investigated whether this level would be consistent with protection at birth. Previous studies have reported that a hemagglutination inhibition (HAI) titer of > 1:80 corresponds to protection (4) and that HAI and ELISA titers are highly correlated (33). Our standard positive control was submitted to the Cornell Animal Health Diagnostic Centre for quantification by HAI. This established that an HAI titer of 1:80 corresponded to an endpoint titer of 3,239 in our in-house ELISA. In **Figure 5**, this is highlighted as a dashed line, with samples > 1:80 HAI titer, extrapolated from the ELISA data, falling within the shaded yellow area. These results suggest that 22 (17.2%) puppies were born with sufficient IgG to provide protection against CPV at birth without ingesting any colostrum.

## Discussion

This study presents a comprehensive analysis of MatAb transfer, both transplacental and via colostrum, in a diverse population of client-owned dogs. While colostral MatAb transfer has been evaluated previously (7), quantification of transplacental transfer has been limited to small groups of puppies sampled as neonates (23, 25, 34). To expand this understanding, we provide the first report of placental transfer evaluation through the collection of umbilical cord blood samples. This approach is widely used in human studies (13, 14) but has not previously been reported in dogs. Blood sampling from puppies prior to their first suckling limits the number of samples collected due to the invasive nature of this method and the reduced likelihood of owner consent when testing client-owned animals. We found that quantification of total IgG in cord samples showed results very similar to total IgG titers in pup serum collected before colostrum ingestion; 0.46 mg/ml in cord serum, compared to previously reported ranges of 0.3–1.2 mg/ml in pup serum (23, 25, 34). Furthermore, virus-specific cord IgG titers were 4.5% (CPV-specific) and 6.0% (CDV-specific) of dam IgG titers, closely matching the 5.7% reported by Pollock and Carmichael, despite their study being conducted in research kennels and our cohort comprising client-owned dogs (4). These findings confirm that cord blood collection is a suitable method for studying placental transfer of canine MatAbs and verify that placental transfer contributes a small proportion of MatAb delivery to pups.

Using our valuable cohort of samples, we quantified virus-specific IgG for two important canine viruses (CPV and CDV), as well as total IgG. Evaluation of canine MatAbs using both approaches has not previously been conducted on the same sample set. We observed significantly higher virus-specific IgG titers in colostrum relative to dam serum, which may be further influenced by the reported decrease in dam serum titers at the time of whelping (35). Interestingly, we observed greater transfer rates of virus-specific IgG into colostrum (8.1-fold for CDV, 10.7-fold for CPV) than for total IgG (3.2-fold). These results suggest that virus-specific MatAbs are preferentially transferred into colostrum, representing the first report of this process in dogs. Identification and exploration of this phenomenon in other domestic species remains limited. A single study in cows provided evidence of selective transfer of pathogen-specific IgG into colostrum (3.9-fold transfer for total IgG, 11-fold transfer for bovine respiratory syncytial virus-specific IgG) (36). Together, these findings suggest that virus-specific IgG is favored for transfer.

The mechanisms underlying the transfer of any IgG into canine colostrum remain unclear, which complicates understanding the factors mediating the selective transfer of virus-specific IgG. Although data from other domestic species are also limited, they provide preliminary insights relevant to our canine findings. The neonatal Fc-receptor (FcRn) has been implicated in IgG transfer into colostrum in some species (cattle, mice, and pigs); however, conflicting results indicate that further research is required (19, 37). A role for FcRn in canine IgG transport has been inferred from studies in other species, but direct evidence is lacking. Nonetheless, our data demonstrating the selective transfer of IgG into colostrum support the involvement of a receptor-mediated transport mechanism. We hypothesize that features of virus-specific IgG enhance this transfer by modulating its interaction with the transport receptor, FcRn. This process may be influenced by various characteristics that affect FcRn binding, such as IgG subclass (21) or posttranslational modifications, including Fc-region glycosylation. In cows, IgG1 is the most abundant subclass in colostrum (38), and its transfer is affected by FcRn binding affinity, although additional factors also contribute. In this study, preliminary investigations of IgG subclasses did not reveal changes in the transfer ratio for IgG2 compared with total IgG. We acknowledge that our study was limited by the lack of available reagents to precisely characterize the four canine IgG subclasses (A–D) (21, 22). Evidence for a role of antibody glycans in MatAb transfer comes from studies showing that bovine colostrum contains IgG with higher sialic acid contained pooled serum (36). Moreover, vaccination in humans has been shown to induce different glycans in virus-specific antibodies but not all IgG (39). Future work is required to further understand this process across different species.

In this study, we identified maternal serum IgG titer as the strongest factor influencing the MatAb titers delivered to puppies. This finding is in contrast with a study by Mila et al., who examined 44 dogs in a single breeding kennel and did not identify an association between maternal serum total IgG titer and colostrum IgG titer (7). Interestingly, this earlier study reported a 2.8-fold enrichment of total IgG in colostrum, which is comparable to the 3.2-fold increase in total IgG observed in our cohort. We suggest that the small difference in total IgG and the lack of correlation between maternal IgG titer and colostrum titer in the earlier study were influenced by sampling strategy and timing. All our colostrum samples were collected within 1 h of delivery, either prior to pup suckling or simultaneously during pups’ first suckle from unoccupied teats, whereas Mila et al. obtained samples up to 24 h postpartum. Colostrum composition is known to change rapidly as it transitions to mature milk; in cattle, IgG concentration 1 day postcalving decreases to approximately 60% of the level measured at 0.5 days (36). This rapid decline coincides with intestinal “closure”, when neonates lose the ability to absorb macromolecules such as IgG. Gut closure occurs within 24 h in both dogs and cows (Baker et al., 2025); consequently, even small differences in sampling time may substantially influence colostrum IgG concentrations across studies. In addition, we observed a weaker association between total IgG titers in serum and colostrum than the correlation identified between virus-specific IgG in colostrum and maternal serum, which could contribute to the discrepancy in our findings. Evidence from other species indicates that maternal serum IgG correlates with colostrum IgG in cats (40). Felidae have a similar endotheliochorial placenta to dogs, so similarities in MatAb transfer may be expected. In species with epitheliochorial placentas, the evidence is less clear; a study in horses showed no correlation between maternal serum and colostrum IgG (41), and bovine studies have generated mixed results.

We did not detect any correlation between IgG titers in colostrum and parity, consistent with findings in pigs (42). Interestingly, in cows, parity has been shown to modulate colostral IgG, with higher IgG concentrations observed in dams of increasing parity (43). These differences may reflect species-specific reproductive and breeding practices, including the age at first breeding, which can influence colostrum composition across successive parturitions. Dam age did not appear to influence IgG titers in colostrum or maternal serum, and, as expected, dam body weight did not correlate with IgG titers in colostrum or cord, consistent with prior findings that breed or size does not affect colostral IgG composition (1). Litter size was not shown to influence MatAb titers in our sample set, although only a single litter had fewer than three pups. In this cohort, we also did not detect any association between reported vaccination status and MatAb titers. A limitation of our study cohort is the high proportion of dams with unknown vaccination status (41%), as recruitment of dogs during emergency surgery limited the completeness of the vaccination dataset.

Our finding that colostrum and cord IgG titers correlate with dam IgG titers strongly suggests that the most efficient strategy to enhance protection in pups will be to boost antibody titers in the dam. We demonstrated that a proportion of pups are born with protective titers of CPV-specific antibodies prior to suckling, and our results indicate that this proportion could be increased by ensuring higher titers in dams. In our population, however, 15.9% of the dams lacked detectable IgG titers to at least one highly pathogenic virus, leaving their puppies completely unprotected despite colostrum intake. As vaccinating dogs during pregnancy is currently not recommended by the World Small Animal Veterinary Association vaccine guidelines (17), it is critical to assess vaccination status prior to breeding. Strategies such as titer testing or administering booster vaccinations before breeding may be appropriate if the dam’s vaccination history is uncertain. Ensuring adequate antiviral IgG titers in the dam will support effective placental transfer and the production of IgG-rich colostrum, thereby maximizing the delivery of protective MatAbs to puppies.

Overall, our study supports the dog as a robust comparative model for studying MatAb transfer, particularly with respect to species-specific differences in reproductive biology and maternal factors. Future comparative studies across mammals will be important for identifying conserved mechanisms and determinants of MatAb transfer.

## Data Availability

The original contributions presented in the study are included in the article/**Supplementary Material**. Further inquiries can be directed to the corresponding author.

## Supplementary material

Supplementary Figure 1 CPV-specific IgG2 quantified in dam serum, colostrum, and cord samples. **(A)** Endpoint titers of CPV-IgG2 specific MatAbs were quantified in clinical samples; dam n=29, colostrum n=24, and cord n=26 litters. Transfer ratios between dam serum and colostrum **(B)** and dam serum and cord serum **(C)** are shown. The dotted line in A represents the lower limit of quantification. Significance was determined using Kruskal-Wallis tests with multiple comparisons **(A)**, and Wilcoxon tests (B/C). Asterisks denote statistical significance: *p* < 0.01 (**)*, p* < 0.001 (***), and *p* < 0.0001 (****).

Supplementary Figure 2 Correlation between total IgG titers and biological variables. All values were determined by total IgG ELISA. **(A)** Total IgG (mg/ml) from dam, colostrum, and cord samples plotted against parity. The horizontal bar represents the mean titer of each group. **(B-D)** Total IgG plotted against dam age, dam weight, and litter size, respectively. Nonlinear regression curves were fitted to the data.
