# Supplementary figures and images for: Quantifying maternal antibody transfer to colostrum and cord blood reveals virus-specific selectivity in dogs
Source: Front Immunol. 2026 Jan 14;16:1753521. doi: 10.3389/fimmu.2025.1753521 (PMC12848613; doi:10.3389/fimmu.2025.1753521)

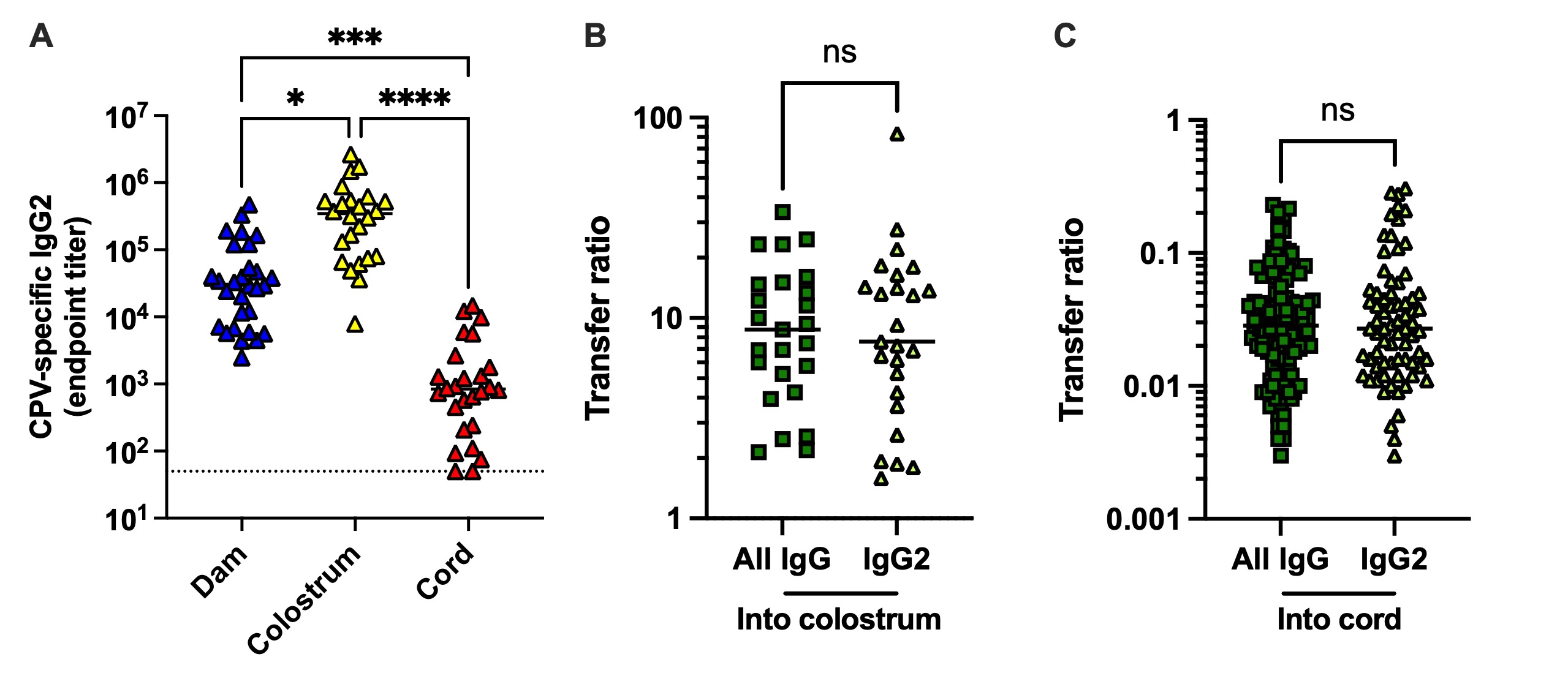

Supplement: Supplementary Figure 1 — CPV-specific IgG2 quantified in dam serum, colostrum, and cord samples. (A) Endpoint titers of CPV-IgG2 specific MatAbs were quantified in clinical samples; dam n=29, colostrum n=24, and cord n=26 litters. Transfer ratios between dam serum and colostrum (B) and dam serum and cord serum (C) are shown. The dotted line in A represents the lower limit of quantification. Significance was determined using Kruskal-Wallis tests with multiple comparisons (A), and Wilcoxon tests (B/C). Asterisks denote statistical significance: p < 0.01 (**), p < 0.001 (***), and p < 0.0001 (****). [file Image1.jpeg]

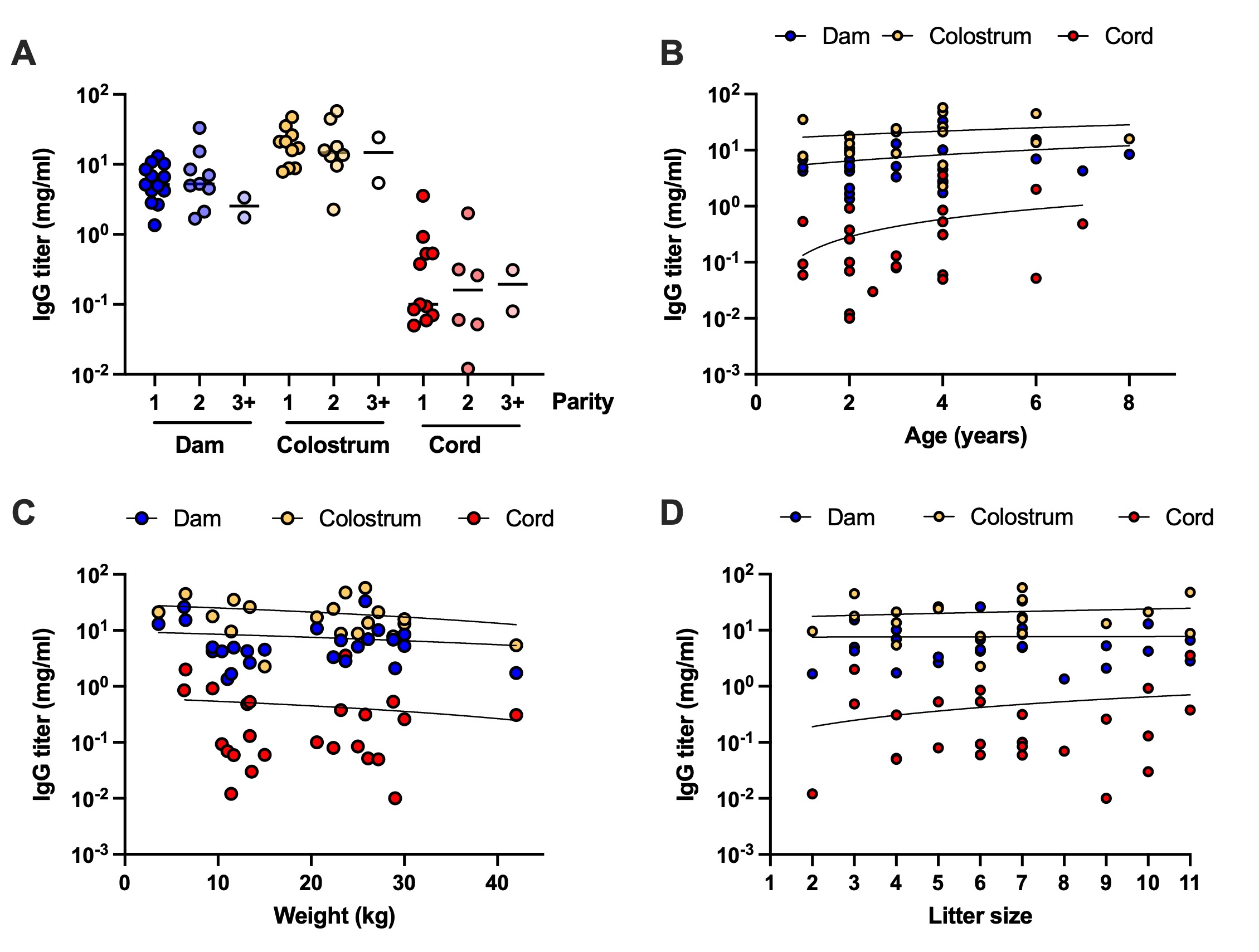

Supplement: Supplementary Figure 2 — Correlation between total IgG titers and biological variables. All values were determined by total IgG ELISA. (A) Total IgG (mg/ml) from dam, colostrum, and cord samples plotted against parity. The horizontal bar represents the mean titer of each group. (B-D) Total IgG plotted against dam age, dam weight, and litter size, respectively. Nonlinear regression curves were fitted to the data. [file Image2.jpeg]
